# Altered Redox Homeostasis in Branched-Chain Amino Acid Disorders, Organic Acidurias, and Homocystinuria

**DOI:** 10.1155/2018/1246069

**Published:** 2018-03-20

**Authors:** Eva Richard, Lorena Gallego-Villar, Ana Rivera-Barahona, Alfonso Oyarzábal, Belén Pérez, Pilar Rodríguez-Pombo, Lourdes R. Desviat

**Affiliations:** Centro de Biología Molecular Severo Ochoa UAM-CSIC, Centro de Diagnóstico de Enfermedades Moleculares (CEDEM), CIBERER, IdiPaz, Universidad Autónoma de Madrid, Madrid, Spain

## Abstract

Inborn errors of metabolism (IEMs) are a group of monogenic disorders characterized by dysregulation of the metabolic networks that underlie development and homeostasis. Emerging evidence points to oxidative stress and mitochondrial dysfunction as major contributors to the multiorgan alterations observed in several IEMs. The accumulation of toxic metabolites in organic acidurias, respiratory chain, and fatty acid oxidation disorders inhibits mitochondrial enzymes and processes resulting in elevated levels of reactive oxygen species (ROS). In other IEMs, as in homocystinuria, different sources of ROS have been proposed. In patients' samples, as well as in cellular and animal models, several studies have identified significant increases in ROS levels along with decreases in antioxidant defences, correlating with oxidative damage to proteins, lipids, and DNA. Elevated ROS disturb redox-signaling pathways regulating biological processes such as cell growth, differentiation, or cell death; however, there are few studies investigating these processes in IEMs. In this review, we describe the published data on mitochondrial dysfunction, oxidative stress, and impaired redox signaling in branched-chain amino acid disorders, other organic acidurias, and homocystinuria, along with recent studies exploring the efficiency of antioxidants and mitochondria-targeted therapies as therapeutic compounds in these diseases.

## 1. Introduction

A growing body of evidence indicates that disruption of redox homeostasis is involved in the pathophysiology of several inborn errors of metabolism (IEMs), including organic acidurias, respiratory chain disorders, fatty acid oxidation disorders, and aminoacidopathies [1–7]. IEMs are defined as genetic defects that affect a metabolic pathway or cellular process and are commonly characterized by a specifically altered biochemical profile that guides the diagnosis [8]. They belong to the category of rare diseases (defined by the EU as those with an incidence lower than 1 in 2000), and most of them are inherited in autosomal recessive fashion. In the classic IEMs, pathology results from accumulation of toxic metabolites and/or from the deficiency or absence of substrates necessary to perform normal cellular functions. Many of them are systemic, and in most cases, they affect the central nervous system [8].

Several IEMs share common pathomechanisms as a consequence of the accumulation of toxic metabolites; these inhibit specific mitochondrial enzymes and processes, resulting in mitochondrial dysfunction, impaired energy metabolism, increased reactive oxygen species (ROS) levels, altered antioxidant response capability, and increased oxidative stress, resulting in damage to DNA, proteins, and lipids [2, 3, 9, 10]. The brain and heart, frequently affected in IEMs, are energy-demanding organs highly sensitive to oxidative stress due to their high rate of oxygen consumption, substantial iron and polyunsaturated lipid contents, and relatively low activity of their antioxidant defences and repair enzymes [11]. Metabolic profiling of oxidative stress in IEM patients' samples has documented the correlation between oxidative stress markers and biochemical and clinical parameters [12, 13].

Mitochondria, which are the major energy producers in the cell, and where many important metabolic pathways take place, are the major ROS producers [14]. Mitochondrial ROS is generated, in the form of superoxide anion (O_2_^−^), at complexes I and III of the electron transport chain and are rapidly converted to hydrogen peroxide (H_2_O_2_) by the enzyme superoxide dismutase (MnSOD). Mitochondrial matrix complexes such as pyruvate dehydrogenase, alpha-ketoglutarate dehydrogenase, or branched-chain alpha-ketoacid dehydrogenase (BCKDH) can also generate O_2_^−^. Other cellular sources of ROS exist, and an active interplay between all of them has been documented [15]. To avoid the noxious consequences of ROS, mitochondria express a variety of antioxidant enzymes, including MnSOD, peroxiredoxins, and glutathione peroxidase (GPX) [16]. Among the nonprotein antioxidants, glutathione (L-*γ*-glutamyl-L-cysteinylglycine) represents the main antioxidant defence present in the cell. An increase in ROS levels correlates with a decrease in reduced glutathione (GSH). Thus, the ratio of oxidized (GSSG) to reduced (GSH) is indicative of cellular redox balance and has been used as a biomarker of oxidative stress in different diseases including IEMs [17–20].

At physiological concentrations, ROS are recognized to act as signaling molecules modifying specific proteins that will transmit a message modulating specific cellular pathways, in what is known as redox signaling [15]. Well-known examples include redox-sensitive changes in conformation and function of receptors, transcription factors, kinases, and phosphatases, which lead to a specific cellular response [15]. At high concentrations, ROS may cause extensive damage to cells, by peroxidation of lipids, carbonylation of proteins, or DNA base oxidation. These toxic effects can disrupt mitochondrial function causing a further increase in ROS production, thus forming a vicious cycle. In cases of chronic, high levels of ROS production, apoptosis or autophagy/mitophagy processes are triggered [16]. All this has led to redefine the concept of oxidative stress, initially conceived as an alteration in the balance between oxidant and antioxidant molecules, to a disturbance in redox signaling with possible molecular and/or cellular damage [15].

In the mitochondria, H_2_O_2_ is the most likely signal involved in redox signaling and in transcription regulation, through thiol/disulfide exchange reactions [21]. Signal transduction by H_2_O_2_ may occur via peroxiredoxin-2 and transcription factor STAT3 [22]. H_2_O_2_ acts on redox-sensitive targets such as transcription factor NRF2 involved in antioxidant response, NF*κΒ* protein complex involved in inflammation, stress kinase JNK, and insulin-like growth factor IGF1, linking energy metabolism and inflammatory responses [23]. Oxidative stress and bioenergetics deficits trigger signaling through mitophagy receptors such as ATG32, BCL2-L-13, and FUNDC1, leading to the elimination of damaged mitochondria [24].

In IEMs, as in cardiovascular and neurodegenerative diseases, the production of pathological amounts of ROS resulting in oxidative damage may indeed disturb redox signaling and induce cell stress responses, which may in some cases lead to cell death [1, 25]. However, the exact molecular mechanisms and cellular pathways involved in this progression are scarcely characterized. In some IEMs, specific activation of stress kinases p38 and JNK and of autophagy/mitophagy and apoptotic pathways has been documented [6, 7, 26, 27].

A recent review provided insight into the adaptive stress responses that operate in IEMs, linking mitochondrial biogenesis to cellular antioxidant, anti-inflammatory, protein quality control, and autophagy functions [1]. As a first line of cellular responses to oxidative stress, the expression of antioxidant enzymes and heat-shock proteins increases, along with an upregulation of glycolysis and activation of mitochondrial biogenesis, as occurs in multiple acyl-CoA dehydrogenation deficiency [28]. ROS-mediated activation of AMP-activated protein kinase (AMPK) could be the link between metabolic reprogramming, mitochondrial biogenesis, and activation of cellular stress repair pathways, followed by the progression to proinflammatory responses controlled by signaling pathways mTOR/HIF-1*α*/NF*κΒ* [1].

The presence of interconnected redox-signaling pathways and their alterations in disease contributing to the pathogenesis suggests that specific compounds targeting excessive ROS or mitochondrial dysfunction may show therapeutic potential. This has been explored in some IEMs, in which several antioxidants or mitochondrial biogenesis activators have proven effective in cellular and/or animal models of disease [29–32]. However, clinical efficacy of these approaches remains unproven. In addition, the possible interference of these therapies with cellular redox signaling should be taken into account for careful design and appropriate clinical testing.

**Figure 1:**
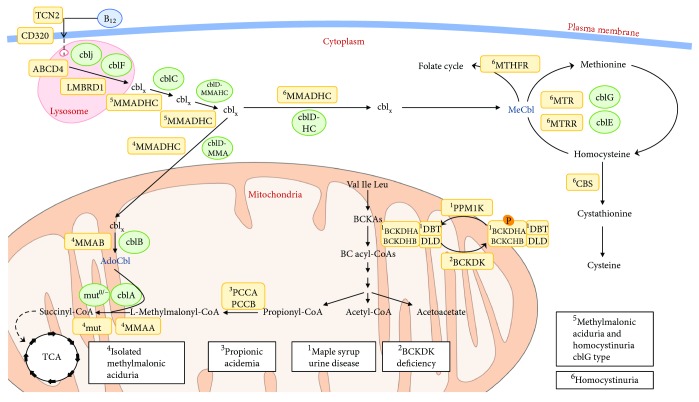
Schematics of the metabolic pathways and their cellular localization affected in branched-chain organic acidurias and homocystinuria disorders mentioned in this work. The affected genes and corresponding diseases (in boxes) are shown for each pathway.

In this study, we describe sources of ROS in specific IEMs; we summarize the available data on altered redox homeostasis and oxidative damage in patients' samples and in animal models of disease; we discuss potential implications for redox signaling and describe the recent advances in antioxidant- and mitochondria-targeted therapies for these pathologies. To this aim, we have focused in selected branched-chain amino acid disorders, organic acidurias, and defects leading to the accumulation of homocysteine (homocystinuria) (Figure 1). For other IEMs, namely inherited mitochondrial diseases and fatty acid oxidation disorders, the readers are referred to recent reviews [9, 33].

## 2. Branched-Chain Amino Acid Disorders and Organic Acidurias

Branched-chain amino acids (BCAAs: Val, Ile, Leu) are essential amino acids not only necessary for protein synthesis but also key nitrogen donors involved in interorgan and intercellular nitrogen shuttling [34] and key nutrient signals involved in important functions including food intake regulation. Leu has long been known as an anabolic nutrient-signaling molecule that stimulates protein synthesis by activation of the mTORC1 pathway in selected tissues [35]. Recently, mitochondrial sirtuin 4 (SIRT4), acting as a lysine deacylase, has been reported to control Leu catabolism and insulin secretion, reinforcing the hypothesis of a possible link between BCAA metabolism and insulin secretion [36]. All these data underscore the critical role of BCAAs metabolism in humans' health and disease, as was evidenced with the characterization of maple syrup urine disease (MSUD) [37] and, more recently, with the description of branched-chain ketoacid dehydrogenase kinase (BCKDK) deficiency [38, 39].

Organic acidurias/acidemias are described as IEMs affecting enzymes, receptors, or transport proteins in the catabolic pathway of amino acids and odd-chain fatty acids and characterized by the abnormal accumulation of organic acids in body fluids [40]. More than 65 organic acidurias have been described, with MSUD, propionic acidemia (PA), and methylmalonic acidurias (MMA) among the most prevalent. A secondary mitochondrial dysfunction induced by accumulating toxic metabolites is an important pathomechanism in these disorders [10]. Patients with organic acidurias show elevated plasma protein carbonyls and lower GSH levels in leukocytes [20]. Here we briefly summarize the published data on branched-chain amino acid disorders and specific organic acidurias with a profile of altered redox homeostasis.

### 2.1. Disorders Involving Branched-Chain *α*-Ketoacid Dehydrogenase

MSUD (MIM #248600), caused by the deficiency of branched-chain *α*-ketoacid dehydrogenase complex (BCKDHc) activity, is characterized by elevated levels of BCAAs and their corresponding *α*-keto-acids (BCKAs) in body fluids and tissues, resulting in complex neurological phenotypes [37]. As the important gatekeeping enzyme that it is, BCKDHc is regulated by reversible phosphorylation catalyzed by a specific BCKD kinase (BCKDK) that inhibits BCKDHc function, halting the catabolic pathway of BCAAs [41], and a dephosphorylation catalyzed by the mitochondrial protein phosphatase PP2Cm (encoded by the *PPM1K* gene) that stimulates BCKDHc activity [42]. Optimal BCKDHc activity necessary to maintain BCAA homeostasis is achieved by the coordinated response of BCKDK and PP2Cm activities. Blockage or unrestrained BCAA metabolism through BCKDHc leads to a dysmetabolism of BCAAs resulting in MSUD or BCKDK deficiency (MIM #614923) (Figure 1), two different clinical conditions with a hallmark of neurological perturbation.

MSUD results from mutations in the genes E1*α*-*BCKDHA* (MIM #608348), E1*β*-*BCKDHB* (MIM #24861), and E2-*DBT* (MIM #248610) [37]. The disease affects 1 : 185,000 newborns worldwide and is manifested by diverse clinical phenotypes, ranging from the most severe form—seen in 70% of patients with MSUD and associated with a profound neurological impact and high mortality if not treated early—to mild forms that present during early development. The mechanisms underlying brain injury are not completely understood. Different studies have been carried out using chemical induction of the disease by BCAAs or BCKAs in cultured cells [43, 44] and animal models [45–48]. All converge in the identification of oxidative stress, brain energy deficit, and/or alterations in the brain's neurotransmission balance, mostly affecting glutamate [46], as important neurodegenerative determinants. Recent studies in human peripheral blood mononuclear cells have shown that 10 mM of BCAAs can directly trigger a mechanism that involves the two major ROS sources, NADPH oxidase and mitochondria. BCAAs stimulate the activation of the redox-sensitive transcription factor NF*κΒ* resulting in the release of proinflammatory molecules [49].

Increases in lipid and protein oxidation are detected in plasma of MSUD patients [50, 51]. The two branched-chain aminotransferases (BCATs) involved in the conversion of BCAAs in BCKAs contain redox-active cysteine residues and could be affected by an oxidative environment. Their inhibition would provoke a further increase in the BCAA levels that could exacerbate the effect of the blockage [34]. An increased inflammatory profile has also been observed in plasma samples from MSUD patients maintained at low BCAA levels indicating the presence of sustained inflammation and activation of the immune system probably as a result of unbalanced ROS production [52].

On the other hand, mutations in the *PPM1K* gene in human result in a mild increase of BCAA and BCKA levels compatible with a mild variant form of MSUD (Figure 1), which nevertheless results in ROS increases along with the activation of the JNK and p38 MAP kinases [42]. This could be related to its critical role for mitochondrial function and cell survival previously described in animal models [53].

In the reverse of the MSUD metabolic problem, BCKDK patients (MIM **#**614923) manifest an unrestrained BCAA metabolism with decreased BCAA and BCKA levels, accompanied by neurocognitive phenotypes [38, 39]. However, also in this case, the mitochondrial response to BCKDK deficiency seems to be the increase in O_2_^−^, alterations in the expression of MnSOD and GPX, and alterations in the bioenergetics' profile with reductions in ATP-linked respiration and intracellular levels. All these data support the hyperfusion response of BCKDK-deficient mitochondria to stress and the altered cell fate observed in patients' fibroblasts [54]. Apart from the canonical ROS-producing sites in mitochondrial electron transfer chain, several reports pointed towards the important role of mitochondrial 2-oxoacid dehydrogenase complexes (2-oxoglutarate dehydrogenase, pyruvate dehydrogenase, and BCKDH) that share the E3 dihydrolipoamide dehydrogenase, as major O_2_^−^/H_2_O_2_ producers under conditions of maximum activities of substrate oxidation [55, 56]. Therefore, the most likely result from the uninhibited activity of BCKDHc could be a misbalance in redox equilibrium and an increase in the production of O_2_^−^/H_2_O_2_ [54–56].

### 2.2. Propionic Acidemia

Propionic acidemia (PA, MIM #606054) is one of the most frequent life-threatening organic acidurias, affecting 1 in 100,000 live births worldwide. PA results from mutations in the *PCCA* or *PCCB* gene that encode the *α* and *β* subunits of propionyl-CoA carboxylase (PCC), respectively. PCC is a biotin-dependent mitochondrial enzyme that catalyzes the reaction of propionyl-CoA to D-methylmalonyl-CoA, the first step of the propionate oxidation pathway (Figure 1). Propionyl-CoA derives from the catabolism of certain amino acids including BCAAs (Ile, Val, Thr, and Met), of cholesterol, and from the beta-oxidation of odd chain fatty acids and bacteria gut production [57]. PCC deficiency results in the accumulation and excretion of propionate, 3-hydroxypropionate, methylcitrate, and propionylglycine, which are the biochemical hallmarks for diagnosis [57]. PA leads to a multisystemic disorder that affects the cardiovascular, gastrointestinal, renal, nervous, and immune systems [58, 59]. The clinical outcome varies from a severe neonatal form characterized by ketoacidosis, feeding refusal, lethargy, failure to thrive, seizures, and encephalopathy to a milder-late onset form with metabolic decompensations, failure to thrive, and diverse neurological deficiencies [60–62]. Cardiomyopathy and neurological features are associated with a high morbidity and mortality and have been attributed to bioenergetic failure and intracellular accumulation of toxic metabolites [63, 64].

A number of *in vitro* and *in vivo* studies of propionyl-CoA metabolites have shown inhibition of enzymes involved in energy production pathways, such as respiratory chain complex III [65] and pyruvate dehydrogenase complex [66]. Furthermore, propionyl-CoA reacts with oxaloacetate to produce methylcitrate that inhibits enzymes such as phosphofructokinase aconitase and citrate synthase [67]. In addition, propionate, at concentrations similar to those found in the plasma of PA patients, strongly inhibits oxygen consumption as well as oxidation of pyruvate and alpha-ketoglutarate in rat liver mitochondria [66, 68]. Moreover, the lack of PCC that blocks the anaplerotic biosynthesis of succinyl-CoA from propionyl-CoA may result in diminished TCA cycle activity [69]. This could be especially relevant for the heart and skeletal muscle, for which propionate is a major anaplerotic substrate. Propionic acid was shown to stimulate the production of O_2_^−^ in the presence of Ca^2+^ influx activators in human neutrophils [70], to increase protein carbonylation in rats [71] and to stimulate lipid peroxidation in rat cerebral tissues [72].

The secondary mitochondrial dysfunction in PA is manifested as a decrease in ATP and phospho-creatine production, a decrease in the activity of respiratory chain complexes, mtDNA depletion, and abnormal mitochondrial structures present in PA patients' biopsies. This is evident particularly in high-energy-demanding organs such as the brain and heart [73–75]. In addition, evidence of oxidative stress and cellular damage has been shown in PA patients' fibroblasts through detection of elevated intracellular H_2_O_2_ levels correlating with the activation of the JNK and p38 pathways [26]. Urinary samples from PA patients show high levels of oxidative stress markers [12].

Alterations in redox homeostasis and mitochondrial function were observed in a hypomorphic mouse model of PA (*Pcca*^−/−^ (A138T)) [5]. These include increased O_2_^−^ production and *in vivo* mitochondrial H_2_O_2_ levels, mtDNA depletion, lipid oxidative damage, and tissue-specific alterations in the activities of OXPHOS complexes and in antioxidant defences. An increase in the DNA repair enzyme 8-guanine DNA glycosylase 1 (OGG1) induced by oxidative stress was also found in the liver of PA mice [32]. These alterations show a good correlation with the altered mitochondrial function and oxidative damage detected in PA patients' samples [12, 73–77].

### 2.3. Methylmalonic Acidurias

Methylmalonic acidurias (MMA) include a heterogeneous group of autosomal recessive genetic disorders caused by deficiency of the mitochondrial enzyme methylmalonyl-CoA mutase (MCM) or by defects in cobalamin (cbl) absorption, intracellular cbl trafficking, or synthesis of adenosylcobalamin (Adocbl) and methylcobalamin (Mecbl), which serve as cofactors for MCM and for the cytosolic enzyme methionine synthase, respectively (Figure 1). Two clinical entities have been described: (i) isolated MMA, characterized by methylmalonic acid accumulation and caused by MCM deficiency, defects in Adocbl synthesis, and methylmalonyl-CoA epimerase deficiency and (ii) combined MMA and homocystinuria, characterized by methylmalonic acid and homocysteine accumulation and caused by defects in intracellular cbl metabolism (Figure 1).

#### 2.3.1. Isolated MMA

MCM (MIM #251000; *mut*^0^ or *mut*^−^ enzymatic subtypes), encoded by the *mut* gene, is a mitochondrial matrix enzyme that catalyzes the conversion of L-methylmalonyl-CoA to succinyl-CoA [57]. Biochemically, the disease is characterized by accumulation of methylmalonic acid and by accumulation of propionate, 3-hydroxypropionate, and 2-methylcitrate, due to activation of alternative pathways of propionate oxidation. Clinical presentation varies from a severe, early onset form of the disease, with neonatal ketoacidosis, lethargy, failure to thrive, encephalopathy, and hepatomegaly, to a milder late-onset presentation usually diagnosed during infancy that has a less serious neurological outcome [57]. Neurological symptoms, including psychomotor delay, irritability, lethargy, hypotonia, convulsions, and coma, have been related to a failure in mitochondrial oxidative metabolism. Mitochondrial dysfunction could be due to a combination of the inhibition of specific enzymes and transporters, limitation in the availability of substrates for mitochondrial metabolic pathways, and oxidative damage [78].

Mitochondrial toxicity of methylmalonic acid has been examined *in vitro* using a variety of normal tissues and extracts, including rat brain, mouse muscle, and bovine heart, suggesting an impairment of mitochondrial function, ROS generation, and other secondary metabolic perturbations [72, 79–81]. In addition, 2-methylcitric acid, malonic acid, and propionyl-CoA inhibit respiratory chain and tricarboxylic cycle [82]. In cultured neurons, methylmalonic acid decreases ATP/ADP ratio, collapses ion gradients, causes membrane depolarization, and increases intracellular calcium levels, leading to necrotic and apoptotic cell death [78].

Experimental evidence using patients' samples and a *mut*-knockout mouse identified multiple oxidative phosphorylation (OXPHOS) defects in MMA perturbations in antioxidant defences and resulting oxidative damage, indicating deficient energy metabolism caused by methylmalonyl-CoA accumulation and reduced succinyl-CoA levels [73, 83–86]. Proteomic analysis of patient liver samples by two-dimensional differential in-gel electrophoresis (2D–DIGE) and mass spectrometry (MALDI-MS and LC-MSMS) showed alterations of enzymes involved in free radical scavenging [87]. In patient's fibroblasts, proteomic analysis (2-D DIGE/MALDI-TOF) identified several differentially expressed proteins related to oxidative stress and apoptosis (cytochrome c, MnSOD, and mitochondrial glycerophosphate dehydrogenase) [88]. In a study using high-dimensional flow cytometry, patients with MMA showed lower GSH levels in T lymphocytes, monocytes, and neutrophils, compared to healthy controls [20]. Oxidative damage markers (F-2 isoprostanes and dityrosine) were found increased in urinary samples from MMA patients [12, 89]. Plasma from patients showed significantly higher levels of protein carbonyls as compared to healthy controls [20].

In addition, various parameters of oxidative stress and apoptosis were evaluated in cultured fibroblasts from a spectrum of patients with MMA showing a significant increase in intracellular ROS content, MnSOD protein and apoptosis rate compared to controls [6]. Furthermore, cytochrome c oxidase deficiency was observed in renal tubular megamitochondria from a MMA patient [90], and mitochondrial ultrastructural abnormalities were found in renal samples from MMA mice [89] and in liver samples from MMA *mut*^0^ patients undergoing transplantation [91].

#### 2.3.2. MMA Combined with Homocystinuria

Cobalamin deficiency type C with methylmalonic aciduria and homocystinuria (cblC; MMACHC; MIM #277400 and #609831) (Figure 1) is the most frequent genetic disorder of vitamin B_12_ metabolism. MMACHC protein is a processing enzyme involved in an early cytoplasmic step in the cofactor-trafficking pathway. It exhibits unusual chemical versatility with demonstrated gluthatione transferase, reductive decyanase, and aquocobalamin reductase activities and converts incoming cobalamin derivatives to an intermediate that can be partitioned into Mecbl and Adocbl synthesis to support cellular needs [92–94]. MMACHC catalyzes the glutathione- (GSH-) dependent dealkylation of alkylcobalamins and the reductive deacyanation of cyanocobalamin [95].

Early-onset patients with cblC disease present multisystem failure while there are late-onset patients exhibiting a milder form, with progressive neurological symptoms and behavioural disturbances [96]. Maculopathy and abnormal vision are extremely common in early-onset patients [97].

Increasing evidence provides support that oxidative stress is closely associated with the physiopathology of this disorder. In this disease, there is a combined contribution of mitochondrial ROS due to accumulation of methylmalonic acid, as well as of homocysteine-derived ROS (see below, the Homocystinuria section). Patient-derived fibroblasts showed elevated ROS, apoptosis, and active phosphorylated forms of p38 and JNK stress-kinase levels [98, 99]. High levels of oxidative damage biomarkers have been found in urinary samples [12]. Decreased levels of proteins involved in cellular detoxification (members of the peroxiredoxin family and isoforms of glutathione-S-transferases) were detected in patient-derived fibroblasts by proteomics techniques [100, 101]. Another proteomic study in patients' lymphocytes revealed the deregulation in the expression of proteins involved in oxidative stress and cellular detoxification, especially those related to glutathione metabolism [102]. In relation to this, an imbalance of glutathione metabolism was observed in *cblC* blood samples with a significant decrease in total and reduced glutathione, along with an increase in different oxidized glutathione forms [19]. GSH depletion could be the consequence of a reduced synthesis since total glutathione and its rate-limiting precursor (cysteine) is decreased in *cblC* patients [19]. In addition, increased levels of protein-bound glutathione were detected, providing evidence of the impact of changes in glutathione pools in the pathogenesis of *cblC* disease through redox regulation of protein function [19]. Furthermore, two mutant MMACHC proteins exhibited impaired glutathione binding, and stabilization of the cob(II)alamin derivative has been observed in presence of physiologically relevant glutathione concentrations. The preferential stabilization of cob(II)alamin by the mutants with a concomitant increase in GSSG production offers insights into ROS production [103].

### 2.4. Glutaric Aciduria Type I

Glutaric aciduria type I (GAI, OMIM #231670) is caused by the deficiency of the mitochondrial enzyme glutaryl-CoA dehydrogenase (GCDH), responsible for the oxidative decarboxylation of glutaryl-CoA to crotonyl-CoA, in the catabolic pathways of lysine and tryptophan [104]. The deficiency causes accumulation of glutarate and 3-hydroxyglutarate, and patients are at risk of acute striatal injury during encephalopathic crises before 4 years of age, frequently precipitated by nonspecific illnesses [105]. These episodes lead to the appearance of neurological symptoms including dystonia, dyskinesia, seizures, and coma [106]. Current treatment for GAI includes dietary lysine restriction and carnitine supplementation, although this does not prevent striatal degeneration in about one-third of the patients. Excitotoxicity, oxidative stress, and mitochondrial dysfunction induced by accumulating metabolites have been associated with brain pathogenesis, although the exact underlying mechanisms remain unclear [4, 107, 108].

Glutaric acid administration induces lipid peroxidation and alterations in antioxidant defences in rats [109]. GCDH-deficient knockout mice (*Gcdh^−/−^)* show a biochemical phenotype comparable to GAI patients but do not develop striatal degeneration [110]. These mice exhibit protein oxidative damage and reduction of antioxidant defences in the brain when subjected to an acute lysine overload [111]. Indeed, GCDH knockout mice fed on a high-lysine chow represent an animal model of GAI encephalopathy g [112]. In these mice, high concentrations of glutaric acid within neurons were correlated with mitochodrial swelling and biochemical changes (depletion of alpha-ketoglutarate and accumulation of acetyl-CoA) consistent with Krebs cycle disruption [113].

### 2.5. Other Organic Acidurias

There are several other organic acidurias in which there is an inhibition of energy metabolism attributed to accumulation of toxic metabolites in mitochondria. These include 3-methylcrotonyl-CoA carboxylase deficiency (MCC, MIM #210200 and #210210), 2-methyl-3-hydroxybutyric aciduria (MOHBA, MIM #203750), 3-methylglutaconic aciduria (MHGA, MIM #250950), D-2-hydroxyglutaric aciduria (D-2-OHGA, MIM #600721), L-2-hydroxyglutaric aciduria (L-2-OHGA, MIM #236792), 3-hydroxy-3-methylglutaric aciduria (3-HMGA, MIM #246450), and ethylmalonic encephalopathy (EE, MIM #602473). Deficiencies in several respiratory chain complexes have been reported in some of these pathologies, such as MOHBA (I and IV) [114], MGA (I and V) [115], D-2-OHGA (V and IV) [116], and EE (IV and II) [117]. Abnormal structure of mitochondria was also found in patients' samples with MHGA [118].

A growing body of experimental evidence indicates that impairment of redox homeostasis induced by the major organic acids accumulating in 3-HMGA contributes to the brain and liver pathophysiology [3]. Lipid and protein damage, along with a decrease of antioxidant defences, have been observed in patients' plasma and urine [3].

A recent transcriptome study in MCC-deficient patient-derived fibroblasts revealed an altered gene expression profile indicative of mitochondrial dysfunction, decreased antioxidant response, and disruption of energy homeostasis [119]. This correlates with previous *in vitro* studies in the cerebral cortex of young rats in which the accumulated metabolites have been shown to disrupt energy homeostasis and cause lipid peroxidation [120].

## 3. Homocystinuria

In several IEMs, increased ROS causes pathophysiological oxidative damage that is not originated in the mitochondria. This is the case of homocystinuria in which excess homocysteine (Hcy) directly promotes ROS formation. Hcy is a sulphur-containing amino acid derived from the demethylation of the essential amino acid methionine. Autoxidation of two Hcy molecules yields the oxidized disulphide homocystine, two protons and two electrons, generating ROS in the form of O_2_^−^, hydrogen radical, or H_2_O_2_ [121]. Formation of mixed disulphides also contributes to the additional formation of ROS [121]. In addition to its inherent reactivity, specific mechanisms such as reduction in bioavailable nitric oxide and decreased glutathione peroxidase activity may play a role in Hcy-induced oxidative stress [122, 123]. Plasma Hcy has been known for decades as a risk marker for cardiovascular disease, which is explained by Hcy-induced oxidative damage in endothelial cells [124]. Induction of NADPH oxidase or downregulation of thioredoxin expression have been postulated as underlying mechanisms [125]. Elevated homocysteine concentration has also been proposed as a risk factor for neurodegenerative diseases inducing neurological dysfunction via oxidative stress [126].

Hcy metabolism stands at the intersection of two pathways: remethylation to methionine and transsulfuration to cystathionine [127]. Inherited homocystinurias have in common accumulation of homocysteine with subsequent neurotoxicity. This group of diseases encompasses two distinctive clinical entities: classical homocystinuria due to cystathionine *β*-synthase (CBS) deficiency (transsulfuration pathway) and the rare inborn errors of cobalamin and folate metabolism (remethylation pathway) [128] (Figure 1).

### 3.1. Homocystinuria due to Cystathionine *β*-Synthase Deficiency

Deficiency of cystathionine *β*-synthase (CBS, MIM #236200) is a disorder of methionine metabolism leading to elevated methionine and an abnormal accumulation of Hcy and its metabolites (homocystine, homocysteine-cysteine complex, and others) in the blood and urine. In the case of excess cellular methionine levels, the transsulfuration pathway plays an important role in Hcy metabolism and converts Hcy into cystathionine with the help of the enzyme CBS (Figure 1). The cystathionine formed is then converted into cysteine by cystathionine *γ*-lyase. Although the pathophysiology of CBS deficiency is not yet well established, Hcy excess or cysteine deficiency rather than the accumulation of methionine is more likely to cause the pathogenesis of this disease [127]. CBS deficiency is clinically characterized by heterogeneous clinical manifestations in various organs and tissues, such as thinning and lengthening of the long bones, osteoporosis, dislocation of the ocular lens, thromboembolism, and mental retardation [127].

Oxidative stress may play an important role in the pathophysiology of homocystinuria, as deduced from studies carried out in patients and animal models [129]. Protein, lipid, and DNA oxidative damage and decreased antioxidant defences have been found in patients [130–132]. Increases in lipid, protein, and DNA oxidative damage biomarkers have been reported in CBS-deficient mice [133] and in samples from animal models subjected to Hcy administration [134, 135].

### 3.2. Homocystinuria due to Remethylation Defects

Remethylation disorders are due to deficiencies of enzymes involved in the remethylation of Hcy to methionine causing homocystinuria. They include defects in methionine synthase (MTR, MIM #156570), methionine synthase reductase (MTRR, MIM #602568), MMADHC 5(MIM #611935), and 5,10-methylene tetrahydrofolate reductase enzyme (MTHFR, MIM #236250) (Figure 1) [136]. Biochemically, these disorders are characterized by Hcy elevation and low-to-normal methionine levels, without urinary methylmalonic acid excretion. Patients present severe clinical symptoms, which are mainly neurological for MTHFR deficiency and neurohematological for MTR and MTRR defects [136]. Two major mechanisms have been proposed to explain the pathogenesis of these diseases: (i) defective methionine synthesis (with a consequent drop in S-adenosylmethionine production and deficiencies of numerous methylation reactions leading to hypomyelination in the central nervous system) [137] and (ii) Hcy excess [127].

Patient-derived fibroblasts with defects in MTR, MTRR, or MTHFR show a significant increase in ROS content, in MnSOD levels, in the rate of apoptosis, and in the levels of the active phosphorylated forms of p38 and JNK stress kinases [7]. Increased mRNA and protein levels of Herp, Grp78, IP_3_R1, pPERK, ATF4, CHOP, asparagine synthase, GADD45, and MAM-associated proteins were also found in these cells indicating an increase in endoplasmic reticulum stress and subsequent mitochondrial calcium overload. In addition, an activation of autophagy process and a substantial degradation of altered mitochondria by mitophagy were detected in patient-derived fibroblasts [138].

## 4. Challenges in ROS Detection

ROS production plays an important role in physiological and pathological processes. In order to understand how they contribute to these cellular processes, it is essential to identify the specific reactive species produced, levels, and subcellular localization. ROS lifetime ranges from nanoseconds to seconds due to their high reactivity and depends on numerous clearance mechanisms such as the intracellular antioxidant levels [139]. Steady state concentration of mitochondrial O_2_^−^ and H_2_O_2_ have been estimated to be in the picomolar and low nanomolar ranges, respectively. ROS detection thereby requires specific molecular probes that promptly interact with ROS to compete with antioxidants and generate stable products, which can be accurately quantified. However, their very short life span and extremely low concentration and instability, as well as the large diversity of possible chemical reactions, make ROS assessment challenging. Excellent reviews by different authors cover the limitations, progress, and perspectives of the most widely used tools and new approaches described for monitoring ROS detection [139–142]. In this section, we focus on some of the most common detection strategies used to asses oxidative stress *in vitro* and *in vivo* in organic acidurias, pointing out some of their methodological constrains and concerns.

Assessment of ROS levels has largely relied on the detection of end products [143]. The small cell-permeable probes 2′,7′-dichlorodihydrofluorescein (H_2_DCF) and dihydroethidium (DHE) are the most commonly used molecules for intracellular ROS detection of H_2_O_2_ and O_2_^−^, respectively. Despite the fact that H_2_DCF is one of the most commonly used probes, many concerns have been raised regarding its mechanism of action and specificity [144]. For instance, it is known that a host of ROS species such as nitric oxide, peroxynitrite anions (ONOO−), and even organic hydroperoxides can oxidize H_2_DCF. In addition, redox reactions with DCF and DCFH may lead to generation of the DCF-free radical anion [145], which produces O_2_^−^ and reacts with antioxidants such as thiols and ascorbate [146]. Intracellular ROS generation throughout oxidation of H_2_DCF has been detected in PA, cobalamin disorders (cblB, cblC, cblE, and cblG), MTHFR deficiency, and MSUD patient-derived fibroblasts [26, 42, 98]. Recently, mitochondrial ROS generation has been measured in the cortex of a mouse model of MMA (induced by MMA and ammonia) [147]. DCF measurements should be carefully considered, and extra controls for the assessment of ROS production are required. Thus, DCF has been suggested as a general oxidative stress indicator instead of serving as a specific proof of H_2_O_2_ production.

DHE has been extensively used for intracellular O_2_^−^ measurements together with its mitochondrial-targeted analog (MitoSOX). The specificity of DHE and its oxidative products has been a matter of debate for decades [144]. DHE can form two fluorescent products. One is ethidium, which is formed by nonspecific redox reactions, while the other is 2-hydroxyethidium (2-OH-E+), a specific adduct of O_2_^−^ [148]. The fluorescent spectra of ethidium and 2-OH-E+ overlap, and it is therefore difficult to accurately measure only 2-OH-E+ by simple fluorescent detection methods. However, careful use of specific wavelengths of excitation allows separation of these to signals [149]. DHE staining has been used to measure O_2_^−^ production in brain and heart cryosections of *Pcca*^−/−^ (A138T) mice [5]. MitoSOX has been used to measure the basal production of mitochondrial O_2_^−^ in cobalamin disorders (cblB and cblE) and in BCKDK-deficient patient-derived fibroblasts [6, 7, 54]. However, the combination of DHE and analytical methods such as high-pressure liquid chromatography and mass spectrometry is essential to validate DHE as a technique to quantify O_2_^−^ [150].

In order to have a better understanding of ROS mitochondrial regulation and its role in oxidative damage and redox signaling, new approaches to measure mitochondrial ROS levels within living organisms are essential. Logan et al. developed a radiometric mass spectrometry probe approach. This method is based on the use of the MitoB probe, which comprises a triphenylphosphonium (TPP) cation driving its accumulation inside the mitochondria, conjugated to an arylboronic acid that reacts with H_2_O_2_ to form a phenol MitoP. Once MitoB is administrated to the animal, the probe is modified *in vivo* by ROS to generate an exomarker product (MitoP). The MitoB/MitoP ratio allows assessment of the levels of ROS *in vivo* [151, 152]. This technique has been applied to the mouse model of PA, to measure relative levels of H_2_O_2_ in the liver and heart [5]. The main advantages of this approach are its high selectivity, *in vivo* localization, and the use of analytical techniques such as LC-MS/MS to identify and quantify exomarkers. However, some significant weaknesses are also observed such as its inherently invasive methodology and the time-consuming nature of sample preparation.

Despite the progress achieved in the field of ROS detection, there is a clear need to develop noninvasive, *in vivo*, and more selective methods to address many important questions related to the role of altered redox homeostasis in the pathology of IEMs.

## 5. Redox-Based Therapeutic Approaches

Most IEMs in which altered redox homeostasis and oxidative damage have been documented as described in this work do not have an effective treatment. Therapy mainly consists on protein restriction and supplementation with special formulas that provide the necessary nutrients for adequate growth and development, as well as the administration of specific compounds aiming to detoxify the accumulated metabolites or to enhance residual enzymatic activities [10, 153]. However, the clinical outcome overall remains unsatisfactory, as multiorgan complications (neurological, cardiac, renal, hematological, and gastrointestinal, among others) persist. In this scenario, antioxidants and mitochondrial activators/protectors may be beneficial to prevent or ameliorate oxidative damage contributing to organ pathophysiology.

**Figure 2:**
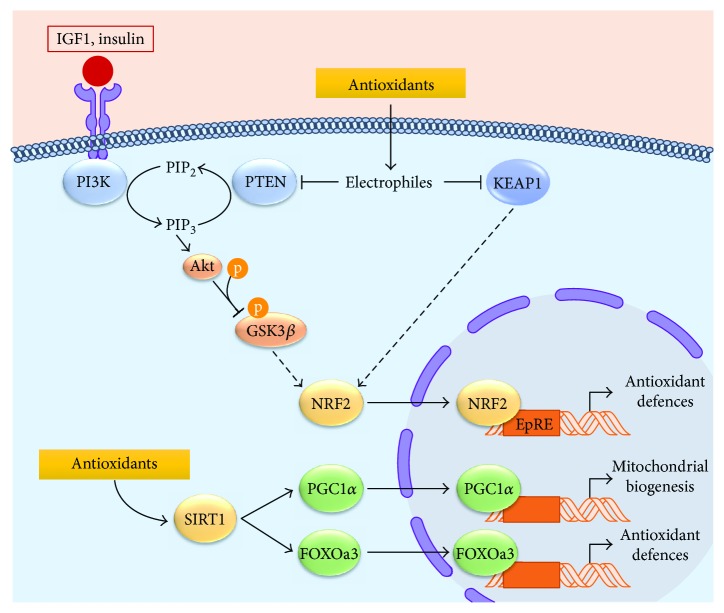
NRF2- and SIRT1-signaling pathways targeted by antioxidant compounds. Under normal homeostatic conditions, NRF2 transcriptional activity is inhibited by KEAP1 and PTEN. KEAP1 directly interacts with NRF2 and mediates its ubiquitination and subsequent proteasomal degradation. Furthermore, PTEN eliminates 3-phosphoinositides (PIP_3_) required for AKT activation, thus leading to GSK3*β* activation and NRF2 phosphorylation. *In vivo* antioxidants act as electrophiles that modify and inhibit KEAP1 and PTEN. When KEAP1 is oxidized, its interaction with NRF2 is disrupted and NRF2 half-life increases. Electrophiles also inhibit the redox-sensitive phosphatase PTEN, allowing PIP_3_ accumulation, AKT activation, and GSK3*β* inactivation. In these conditions, NRF2 is translocated to the nucleus where it binds to the electrophile response element (EpRE) and drives the expression of antioxidant defence genes. Additionally, antioxidant compounds modulate SIRT1 pathway favouring the antioxidant defence response and mitochondrial biogenesis by activation of FOXOa3 and PGC1*α* transcription factors, respectively.

Chemically, antioxidants are reduced compounds that directly react with ROS avoiding the oxidation of a third molecule. The *in vitro* mode of action of nutraceutical and synthetic antioxidants is mainly based on their capacity to act as free radical scavengers. However, *in vivo* antioxidant response depends on distribution, absorption, metabolism, and excretion of the compound, hampering a direct action on ROS. In fact, it is now clear that *in vivo* antioxidants provide indirect cellular and tissue protection against oxidative damage by targeting NRF2 and NAD-dependent protein deacetylase sirtuin-1 (SIRT1) [154, 155], modulating the antioxidant response (Figure 2).

NRF2 transcription factor regulates cellular homeostasis by binding to regulatory DNA regions known as electrophile response elements or EpRE (previously named antioxidant regulatory elements (AREs)). Under oxidative stress, NRF2 translocates to the nucleus, binds to EpRE sequences, and promotes the transcription of antioxidant enzymes, genes of GSH metabolism, and NADPH regeneration (Figure 2) [156].

Antioxidant response can also be driven by SIRT1, a class I histone deacetylase that mediates deacetylation in a NAD+-dependent manner. SIRT1 is mainly localized in the nucleus where, apart from histones, it can interact with and deacetylate peroxisome proliferator-activated receptor gamma coactivator 1-alpha (PGC1*α*) and Forkhead box protein O3 (FOXOa3) [157, 158]. Deacetylated PGC1*α* and FOXOa3 have an increased transcriptional activity and promote mitochondrial biogenesis and expression of antioxidant genes (Figure 2). Therefore, NRF2 and SIRT1 activation by polyphenolic compounds represent the main targets for antioxidant therapy to reduce oxidative damage.

Several studies have evaluated different antioxidant compounds as adjuvant therapy in a number of IEMs. The brain is highly sensitive to free radicals due to its high energetic demands, its high lipid content, and its low regeneration capacity. In this context, oxidative damage has been proposed as one of the mechanisms involved in the development of the characteristic neurological symptoms of PA, MMA, MSUD, GAI, and homocystinuria patients. Even though the transport rate of antioxidants through the blood brain barrier is low, different studies have reported a reversion of brain damage after antioxidant treatments in these diseases [159–162].

In organic acidurias, a beneficial effect of L-carnitine supplementation with *in vitro* antioxidant properties has been described [13, 163]. L-carnitine shows antioxidant capacities as it can act as ROS scavenger and reduce lipid peroxidation to an extent comparable to that seen with *α*-tocopherol [164]. It also protects endogenous antioxidant enzymes from peroxidative damage and induces elevated cellular GSH [31]. L-carnitine supplementation is routinely included in the treatment of PA and MMA, to prevent a secondary carnitine deficiency and to promote detoxification of propionic and methylmalonic acids, which are excreted in the form of carnitine esters. Evaluation of oxidative stress parameters in urine from PA and MMA patients during treatment with L-carnitine- and protein-restricted diet showed a significant reduction of urinary and plasma biomarkers of oxidative damage to lipids and proteins [13, 163]. L-carnitine treatment was also shown to reduce plasma malondialdehyde concentrations and urine dityrosine and isoprostanes in patients with MSUD [51, 165].

In a MMA patient with optic neuropathy, CoQ_10_ treatment in conjunction with vitamin E resulted in improved visual acuity [166]. Interestingly, treatment of MMA mice with CoQ_10_ and vitamin E showed a significant amelioration in the loss of glomerular filtration rate and a normalization of plasma lipocalin-2 levels [89], indicating that the therapeutic effects are not restricted to the nervous system.

Recently, *in vitro* and *in vivo* studies in PA have also shown the positive effect of antioxidant treatment. Using patients' fibroblasts, different antioxidants (tiron, trolox, resveratrol, and MitoQ) significantly reduced H_2_O_2_ levels and regulated the expression of antioxidant enzymes [30]. In the hypomorphic mouse model of PA [167], oral treatment with resveratrol and MitoQ protected against lipid and DNA oxidative damage and induced the expression of antioxidant enzymes [32]. In addition to its antioxidant properties, resveratrol exerts different effects on mitochondrial function and dynamics [168], which clearly contributes to its beneficial effect in IEMs with mitochondrial ROS production, such as organic acidemias and respiratory chain defects [169].

A clinical trial (NCT01793090) is ongoing in patients with MMA cblC-type defect to investigate the safety and efficacy of the EPI-743 compound in relation to visual and neurological impairment. This molecule targets NADPH quinone oxidoreductase 1, thus providing beneficial effects in diseases characterized by oxidative stress and alterations in glutathione redox balance, as is the case of mitochondrial diseases [170].

Taurine, a compound potentially reducing oxidative damage, is being tested in a clinical trial with homocystinuria (CBS-deficient) patients (NCT01192828). Taurine treatment in a mouse model of CBS deficiency reversed GSH depletion with potential beneficial effects [171].

Although the use of antioxidants as potential therapies holds great interest for many diseases (including IEMs) in which oxidative stress plays a pathophysiological role, there is to date limited clinical evidence of their efficacy, which may be related to pharmacokinetic and/or pharmacodynamics reasons [172]. The intracellular levels of the specific antioxidant, its distribution throughout the body, together with the subcellular location are factors that influence the *in vivo* effects. Moreover, antioxidants react with free radicals with the same rate constant as other natural molecules, and their intracellular concentrations cannot overcome this kinetic limitation. Therefore, antioxidant effectiveness *in vivo* could be limited [154].

Throughout the years, different studies have shown both beneficial and adverse effects of antioxidant compounds. *In vitro β*-carotene shows antioxidant and anti-inflammatory effects when administered at low doses whereas prooxidant and proinflammatory responses have been reported at higher concentrations [173, 174]. Similar contradictory results have been obtained in *in vivo* studies. While in a randomized trial dietary consumption of *β*-carotene and vitamins C and E did not affect cancer incidence and mortality [175], higher doses of these compounds did not show any health beneficial effect. In fact, they could significantly increase the risk of mortality (reviewed in [176, 177]). ROS are required at physiological concentrations for optimal cellular homeostasis [178]. Therefore, free radical scavenging by antioxidants could be undesirable as disruption of redox balance would lead to cellular dysfunction and originate different side effects.

New indirect antioxidant approaches targeting redox enzymes or NRF2 agonists that upregulate cellular antioxidant defences are promising alternatives that warrant further investigation [155].

## 6. Conclusions and Future Perspectives

**Figure 3:**
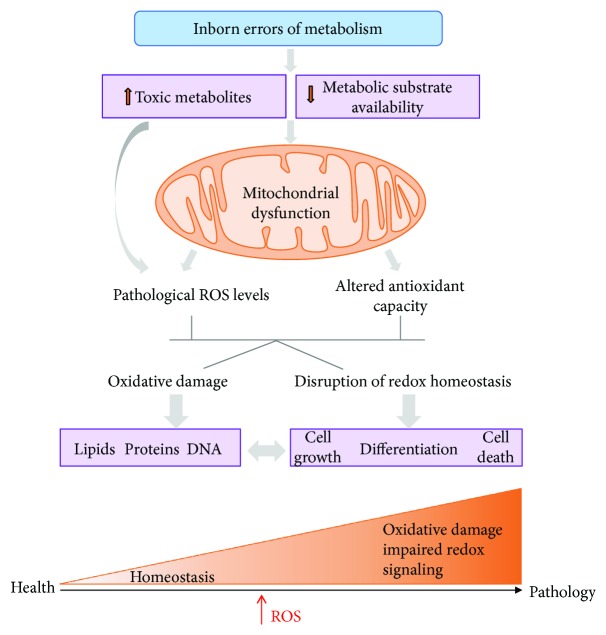
ROS and oxidative damage contribute to IEM physiopathology. The scheme shows the consequences of toxic metabolite accumulation in IEMs on mitochondrial function and ROS increase, which in turn results in oxidative damage and alteration in ROS signaling contributing to disease pathology.

The pathophysiological role of ROS in IEMs has been firmly established in many studies revealing oxidative damage to proteins, lipids, and DNA in patients' samples and animal models, correlating with clinical and biochemical parameters (Figure 3). Consequently, different antioxidants have been successfully used in cellular and animal models of IEMs [30–32, 89, 98]. Despite these positive results, for other diseases, there is no clear conclusion on the efficacy of antioxidant treatments in human clinical trials, which limits the expectations also for IEMs.

In the past years, we have gained deeper insights into the complex and dynamic roles of ROS signals in normal and pathological processes, yet the field of redox signaling in IEMs is still in its infancy. Novel technologies such as redox proteomics [179] or transgenic animals expressing redox reporters [180] are revolutionising the field and represent excellent tools to investigate redox regulatory networks in disease. They may also provide insight to explain the poor performance of antioxidant therapies in clinical trials. In many IEMs, mitochondrial ROS-targeting agents that accumulate in the mitochondria and compounds activating or improving mitochondrial function may constitute a valid approach. Further preclinical research is warranted to investigate basic mechanisms of redox signaling and their implications for IEM disease progression, as well as exploring the benefits of targeted antioxidant therapies.
